# Defective Mitochondrial Dynamics and Protein Degradation Pathways Underlie Cadmium-Induced Neurotoxicity and Cell Death in Huntington’s Disease Striatal Cells

**DOI:** 10.3390/ijms24087178

**Published:** 2023-04-13

**Authors:** Paul J. Kamitsuka, Marwan M. Ghanem, Rania Ziar, Sarah E. McDonald, Morgan G. Thomas, Gunnar F. Kwakye

**Affiliations:** Neuroscience Department, Oberlin College, 119 Woodland Street, Oberlin, OH 44074, USA

**Keywords:** huntington’s disease, cadmium, neurotoxicity, neurodegeneration, bioenergetics, mitochondrial dynamics, autophagy, ubiquitin–proteasome system (UPS)

## Abstract

Exposure to heavy metals, including cadmium (Cd), can induce neurotoxicity and cell death. Cd is abundant in the environment and accumulates in the striatum, the primary brain region selectively affected by Huntington’s disease (HD). We have previously reported that mutant huntingtin protein (mHTT) combined with chronic Cd exposure induces oxidative stress and promotes metal dyshomeostasis, resulting in cell death in a striatal cell model of HD. To understand the effect of acute Cd exposure on mitochondrial health and protein degradation pathways, we hypothesized that expression of mHTT coupled with acute Cd exposure would cooperatively alter mitochondrial bioenergetics and protein degradation mechanisms in striatal ST*Hdh* cells to reveal novel pathways that augment Cd cytotoxicity and HD pathogenicity. We report that mHTT cells are significantly more susceptible to acute Cd-induced cell death as early as 6 h after 40 µM CdCl_2_ exposure compared with wild-type (WT). Confocal microscopy, biochemical assays, and immunoblotting analysis revealed that mHTT and acute Cd exposure synergistically impair mitochondrial bioenergetics by reducing mitochondrial potential and cellular ATP levels and down-regulating the essential pro-fusion proteins MFN1 and MFN2. These pathogenic effects triggered cell death. Furthermore, Cd exposure increases the expression of autophagic markers, such as p62, LC3, and ATG5, and reduces the activity of the ubiquitin–proteasome system to promote neurodegeneration in HD striatal cells. Overall, these results reveal a novel mechanism to further establish Cd as a pathogenic neuromodulator in striatal HD cells via Cd-triggered neurotoxicity and cell death mediated by an impairment in mitochondrial bioenergetics and autophagy with subsequent alteration in protein degradation pathways.

## 1. Introduction

Huntington’s disease (HD) is a fatal neurodegenerative disorder characterized by neuropsychiatric dysfunction, cognitive impairment, and motor incoordination [1]. HD is caused by an expansion in the CAG trinucleotide repeat encoding glutamine within exon 1 of the Huntingtin (*HTT*) gene [2,3]. Wild-type *HTT* is required for embryonic development [4], transcriptional regulation [5], axonal trafficking [6], metal transport and function [7], synaptic transmission [8], and cellular metabolism [9]. Healthy individuals express six to thirty-six CAG repeats; however, HD patients present with greater than thirty-six polyglutamine repeats [10]. Expanded mHTT induces protein misfolding and aggregation resulting in the selective loss of GABAergic medium spiny neurons (MSNs) within the striatum. The striatum is involved in voluntary movement and decision [11]. Although the pathogenic mechanisms underlying HD remain to be fully elucidated, metal dyshomeostasis, transcriptional dysregulation, and mitochondrial dysfunction are central to HD pathogenesis [12,13,14,15,16,17]

Under non-pathogenic conditions, mitochondria maintain a dynamic balance between fission and fusion events to create tubular networks for efficient energy production and distribution [18,19]. mHTT can also promote oxidative stress that disrupts the mitochondrial balance by over-expressing the pro-fission dynamin-related protein 1 (DRP1) and down-regulating pro-fusion proteins, such as mitofusin 1/2 (MFN1/2) and optic atrophy-1 (OPA1) [20]. Increasing expression of fission proteins leads to a shift toward mitochondrial fission resulting in the fragmentation of the mitochondrial networks needed to provide the metabolic requirements of neurons [21,22]. Eukaryotic cells employ two major degradation routes for clearing misfolded proteins and damaged organelles: (1) the ubiquitin–proteasome system (UPS) or (2) the autophagy–lysosome pathway [23,24]. The UPS functions in the cytoplasm and nucleus to facilitate the recycling and degradation of short-lived and misfolded soluble proteins [25]. In contrast, the autophagy–lysosome pathway degrades long-lived proteins and organelles by the formation of double-membrane-bound autophagosomes in the cytoplasm [26,27]. Alteration in either or both pathways contribute to HD pathogenesis [28,29,30,31]. Importantly, mHTT impedes the cellular response to mitochondrial damage by inhibiting the autophagy–lysosome pathways [32], thereby contributing to the accumulation of dysfunctional mitochondrial fragments and the activation of cell death pathways [33].

Despite the genetic etiology of HD, there is accumulating evidence to indicate that environmental factors heavily influence the rate and severity of HD pathogenesis [12,15,34,35,36,37]. A previous study reported that approximately 60% of the variability in the age of onset in the Venezuelan-kindred population was influenced by unknown environmental factors [38]. Additionally, twin studies have reinforced the critical role of an individual’s environment on the severity and progression of HD [39,40,41]. Among the factors influencing HD pathogenesis, metal dyshomeostasis exacerbates HD pathology within in vitro and in vivo models as well as in HD patients [13,42,43,44]. Although metals, such as manganese (Mn), zinc (Zn), iron (Fe), and copper (Cu), are essential to biological function as co-factors [45], their imbalance may induce oxidative stress, cytotoxicity, and neurodegeneration [46]. For example, overexposure to Mn results in Manganism, a Parkinson’s disease (PD)-like neurological disorder [47]. Expression of mHTT alters Mn, Fe, and Cu homeostasis, resulting in its accumulation in the basal ganglia, transcriptional deregulation, oxidative stress, protein aggregation, and neurotoxicity [14,15,48,49]. One heavy metal of increasing concern due to its environmental abundance is cadmium (Cd) [50].

Cd is a ubiquitous heavy metal found in sewage, phosphate fertilizers, nickel–cadmium (NiCd) batteries, plated metal, plastics, and pigments [51]. It is enriched in the tobacco plant [52], and, upon overexposure, Cd can accumulate within the blood, kidneys, liver, reproductive organs, and motor control centers of the brain, specifically the parietal cortex, striatum, and cerebellum [53,54,55]. The long biological half-life of Cd (15–20 years) allows Cd to accumulate and induce metal dyshomeostasis, oxidative stress, mitochondrial dysfunction, and perturb protein degradation pathways, resulting in neurodegeneration [55,56]. Although Cd exposure primarily occurs occupationally, the tobacco plant has been shown to enrich Cd in its leaves. Tobacco smokers have four to five times higher Cd levels compared with non-smokers [57]. Interestingly, the HD population has a higher rate of tobacco smoking than age-matched non-smoker controls [58]. Given the overlap in pathogenic mechanisms between Cd and mHTT, overexposure to Cd may negatively impact the rate of HD pathogenesis. We have previously demonstrated that striatal cells expressing mHTT exhibit greater sensitivity to chronic Cd-induced neurotoxicity compared with healthy wild type (WT) cells, ultimately leading to caspase-mediated cell death via inhibition of extracellular-regulated kinase (ERK), down-regulation of antioxidants, and NADPH oxidase (NOX)-mediated oxidative stress [13].

Recognizing the vital role of mitochondrial function in health and early in HD pathogenesis [59] and the accumulation of Cd in the basal ganglia region [54,60], we examined the effects of acute Cd exposure on mitochondrial bioenergetics, turnover, function and its connection with protein degradation pathways using an established striatal murine cell line ST*Hdh* model of HD. We hypothesize that acute exposure to Cd will aggravate mHTT-induced mitochondrial dysfunction by impairing its respiratory ability and dynamics and disrupting protein degradation machinery.

## 2. Results

### 2.1. HD Cells Are More Sensitive to Early Cd-Induced Neurotoxicity and Exhibit Greater Cell Death

**Figure 1:**
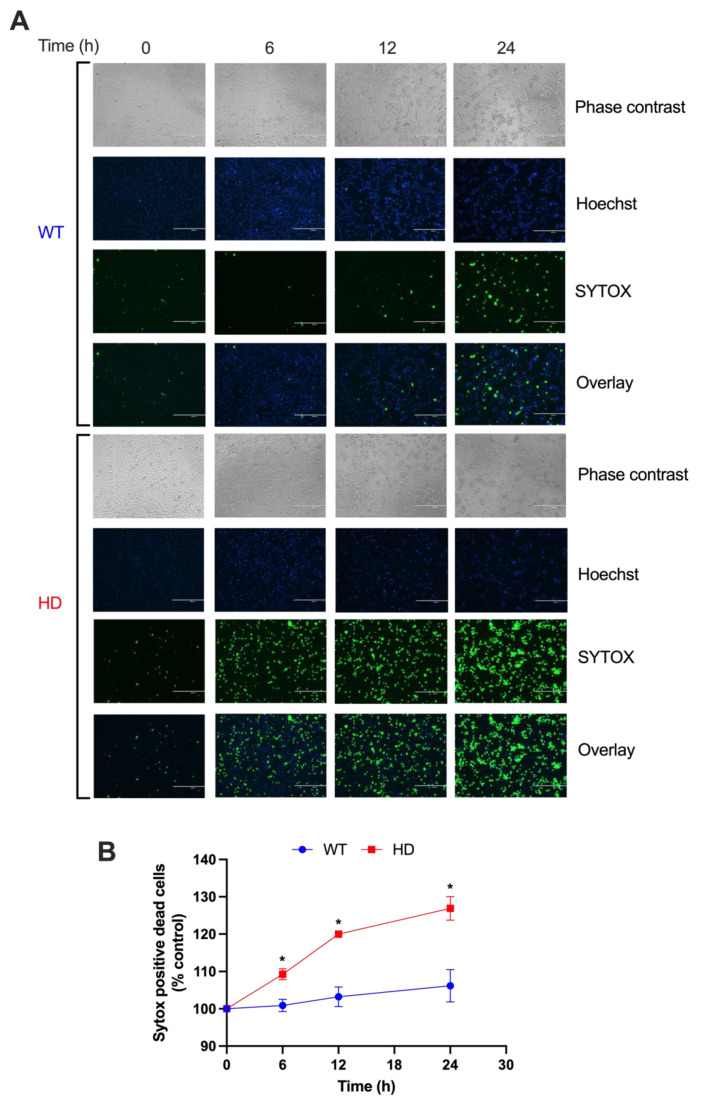
HD striatal cells are more sensitive to Cd-induced cell death compared with WT. (**A**) Representative qualitative images and (**B**) quantitative analyses of cell death in WT and HD cells after exposure to 40 µM CdCl_2_ were examined at 0, 6, 12, and 24 h using an SYTOX Green cell death assay and Hoechst DNA stain. Cell death is expressed as the percentage of the respective vehicle. Results are represented by the mean ±  SEM from four biological replicates (N = 4). Sidak’s multiple comparison test compared the effect of Cd-induced cell death between genotypes at the indicated time points (* *p* < 0.01). Images at 10× magnification.

We have previously reported that exposure to low concentrations of CdCl_2_ (5, 10, 20, and 40 µM) for 48 h significantly increases cytotoxicity in HD striatal cells compared with WT [13]. Given this long duration of exposure, which mimics chronic Cd exposure, we aimed to examine the effects of acute Cd exposure at earlier time points. We chose 40 µM CdCl_2_ and the exposure times between 6 and 24 h to better understand the non-pathogenic and pathogenic effects of Cd in HD. A SYTOX Green cell death assay shows that HD cells exhibit a significant increase in cell death as early as 6 h exposure when compared with their respective vehicle controls or WT cells at the indicated time points, with minimal to no significant increase in cell death within WT cells when compared to its respective vehicle control (Figure 1A,B). A two-way ANOVA reveals a significant effect of exposure time (h) (F(3, 18) = 22.92, *p* < 0.001), genotype (F(1, 6) = 39.81, *p* < 0.001), and exposure time by genotype interaction (F(3, 18) = 9.23, *p* < 0.001), suggesting that each genotype has a unique response to Cd exposure that is dependent on the duration of exposure. Importantly, we observe a significant increase in cell death in the HD cells at 12 h and 24 h above the respective vehicle control.

### 2.2. HD Cells Are More Susceptible to Cd-Induced Mitochondrial Dysfunction Compared with WT

**Figure 2:**
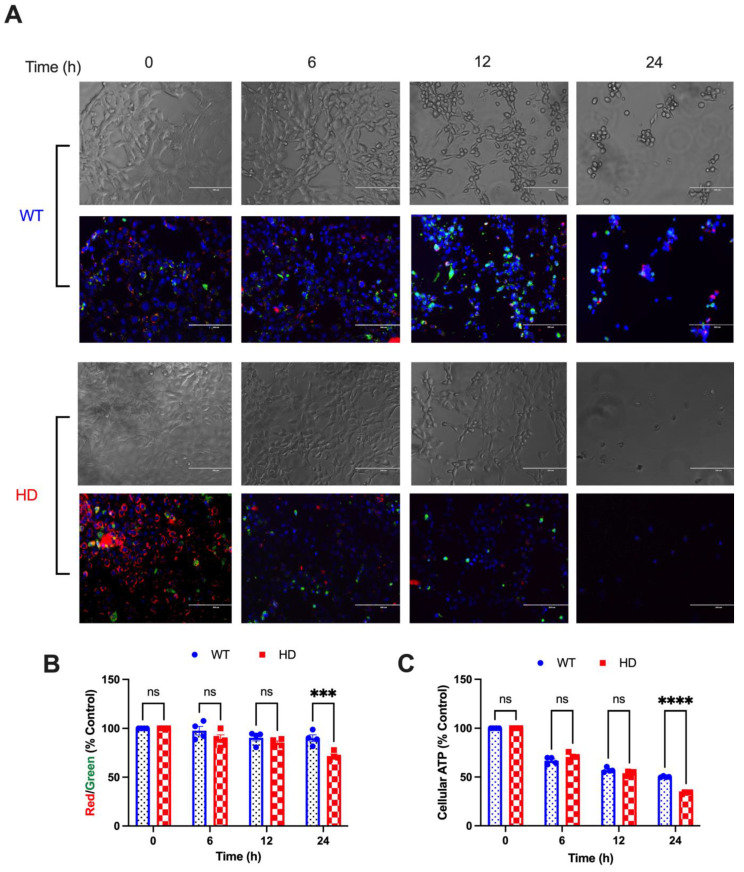
HD striatal cells exhibit reduced mitochondrial bioenergetics compared with WT upon acute Cd exposure. (**A**) Representative qualitative brightfield images of JC-1 dye (red J aggregates and green monomers) and Hoechst DNA stain (blue) staining, and (**B**) quantitative representations of MMP upon exposure to 40 µM CdCl_2_ for 0, 6, and 24 h. MMP is represented as a ratio of red J-aggregates to green monomers normalized to its respective vehicle control. Results are represented by the mean  ±  SEM from four biological replicates (N = 4). (**C**) Cellular ATP measurement in WT and HD cells upon 40 µM Cd exposure at the indicated time points. Results are represented by the mean  ±  SEM from four biological replicates (N = 4). Sidak’s multiple comparison test compared the effect of Cd on MMP and cellular ATP levels between genotypes at the indicated time points (*** *p* < 0.001, **** *p* < 0.0001, Student’s *t*-test). Images at 20× magnification.

The JC-1 assay was used to evaluate changes in MMP in WT and HD cells after 40 µM CdCl_2_ exposure. The ratio of red J-aggregates to green monomers is a measure of mitochondrial membrane potential (MMP). Qualitative analysis reveals an early non-significant reduction in MMP at 6 h in HD cells compared with WT, as depicted by a reduction in red aggregates compared with green monomers (Figure 2A). Quantitative analysis of the ratio of red J-aggregates to green monomers shows a gradual trend in MMP reduction in both WT and HD cells upon Cd exposure. Importantly, 24 h Cd exposure causes a significant decrease in MMP in HD cells compared with WT (Figure 2B). Quantitative analysis of MMP supports the time and genotype effect of Cd on MMP. A two-way ANOVA confirms this effect by revealing a significant effect of genotype (F(3, 18) = 22.57, *p* < 0.001), exposure time (F(1, 6) = 7.908, *p* = 0.0307), and exposure time by genotype interaction (F(3, 18) = 4.975, *p* = 0.0109).

### 2.3. HD Promotes Cd-Induced Alterations in Mitochondrial Fission Proteins

**Figure 3:**
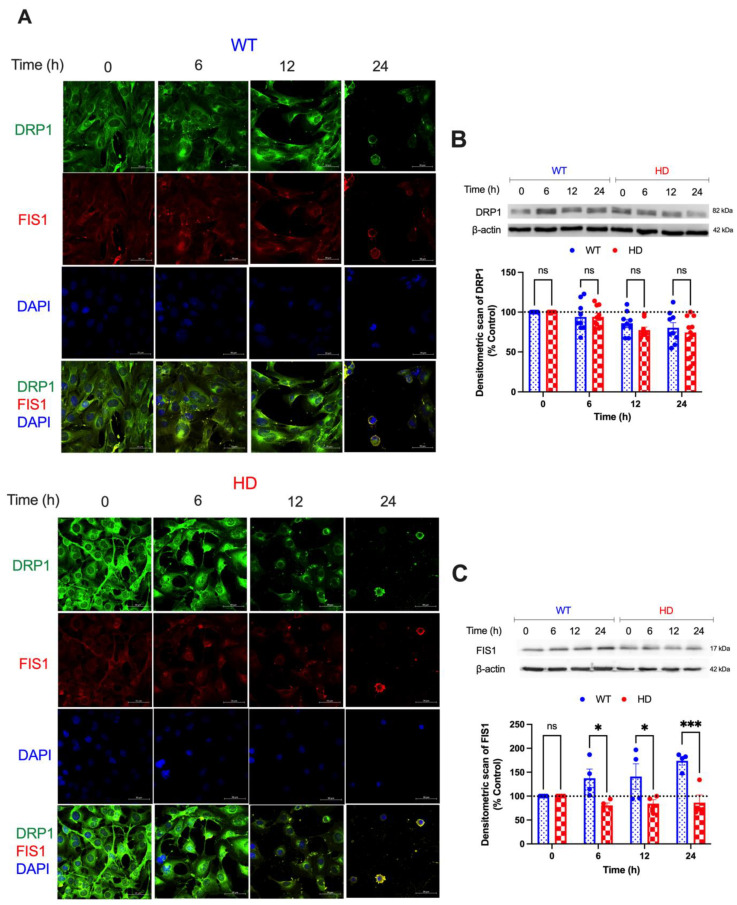
Effect of Cd exposure on the expression of mitochondrial fission proteins in WT and HD striatal cells. (**A**) Representative confocal images show the effect of 40 µM CdCl_2_ exposure on the expression of DRP1 (green) and FIS1 (red) co-stained with DAPI (blue) upon 0, 6, 12, and 24 h exposures. Scale bar: 50 µm. (**B**) Western blot analysis of DRP1 and (**C**) FIS1 protein expression. Quantitative immunoblotting data is represented as percent expression relative to the respective control within each genotype. Representative images of Western blot analysis are shown. Results are represented by the mean  ±  SEM from four to nine biological replicates (N = 4–9). ns indicates no significant difference between genotypes at the indicated exposure time. Sidak’s multiple comparison test compared the effect of Cd on endogenous DRP1 and FIS1 expression between genotypes at the indicated time points (significant differences: * *p* < 0.05 and *** *p* < 0.001, Student’s *t*-test). Sidak’s multiple comparison test compared all treatments back to the vehicle for each genotype. Images at 63× magnification.

To better understand the overall changes in mitochondrial dynamics and function following Cd exposure, we examined the expression of key endogenous proteins that facilitate mitochondrial fission (DRP1 and FIS1) and fusion (MFN1 and MFN2) after 0, 6, 12, and 24 h of Cd exposure. A two-way ANOVA of DRP1 expression reveals a significant effect of exposure time (h) (F(3, 48) = 9.387, *p* < 0.001) and no effect of genotype or genotype by time interaction (Figure 3A,B). Subsequently, we examined the effects of Cd on FIS1 expression, given the role of FIS1 as the adaptor protein of DRP1. As early as 6 h, we observed a significant increase in FIS1 protein expression in WT cells compared with HD following Cd exposure (Figure 3A,C). A two-way ANOVA shows a significant effect of genotype (F(1, 6) = 12.45, *p* = 0.0124) and an exposure time by genotype interaction (F(3, 18) = 5.723, *p* = 0.0062) in FIS1 protein expression.

### 2.4. Mitochondrial Fusion Proteins Are Modulated by Acute Cd Exposure

**Figure 4:**
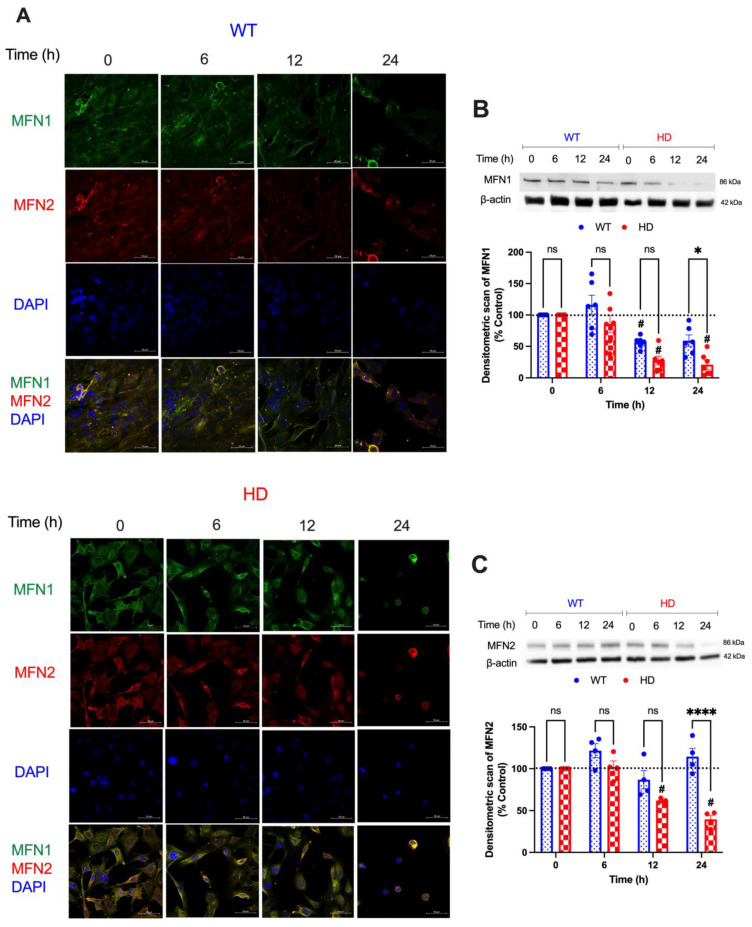
Cd exposure reduces the expression of the pro-fusion mitochondrial protein in HD striatal cells. (**A**) Representative confocal images showing the expression of MFN1 (green) and MFN2 (red) co-stained with DAPI (blue) following 40 µM CdCl_2_ exposure for 0, 6, 12, and 24 h. Scale bar: 50 µm. (**B**) Immunoblotting analysis of MFN1 and (**C**) MFN2 in WT and HD striatal cells exposed to 40 µM Cd for the indicated time points. Quantitative immunoblotting data is represented as percent expression relative to the respective control within each genotype. Representative images of Western blot analysis are shown. Results are represented by the mean  ±  SEM from four to six biological replicates (N = 4–6). Sidak’s multiple comparison test compared the effect of Cd on endogenous MFN1 and MFN2 expression between genotypes at the indicated time points (significant difference: * *p* < 0.05, **** *p* < 0.0001, Student’s *t*-test). ns indicates no significant difference between genotypes at the indicated exposure time. Sidak’s multiple comparison test compared all treatments back to the vehicle for each genotype. # indicates statistical significance (*p* < 0.05) compared with the respective negative controls. Images at 63× magnification.

To complement this change in pro-fission protein expression, we examined the expression of pro-fusion proteins (MFN1, MFN2). A two-way ANOVA reveals a significant effect of genotype (F(1, 10) = 12.68, *p* = 0.0052), exposure time (F(3, 30) = 10.12, *p* < 0.0001), and a time by genotype interaction (F(3, 30) = 3.464, *p* = 0.0284) on MFN1 expression, indicating that HD cells exhibit a progressive and significant reduction in MFN1 expression that is directly related to the duration of Cd exposure. Furthermore, a post hoc analysis with the Student’s *t*-test shows a significant reduction from baseline MFN1 expression in HD cells compared with WT (*p* < 0.05) as early as 6 h (Figure 4A,B). Similarly, we observed a significant reduction in MFN2 expression in HD cells compared with WT by 24 h. A two-way ANOVA highlighted a significant effect of exposure time (F(3, 18) = 20.68, *p* < 0.0001), genotype (F(1, 6) = 19.34, *p* = 0.0046), and time by genotype interaction (F(3, 18) = 16.63, *p* < 0.0001). Post hoc analysis with the Student’s *t*-test revealed a significant decrease (*p* < 0.0005) in MFN2 expression in HD cells compared with baseline at 12 and 24 h. These findings suggest that Cd down-regulates MFN1 and MFN2 expression in a genotype- and time-dependent manner, likely contributing to the perturbation of mitochondrial dynamics and bioenergetics (Figure 4A,C).

### 2.5. Key Autophagic Markers Are Upregulated in HD Cells upon Cd Exposure

**Figure 5:**
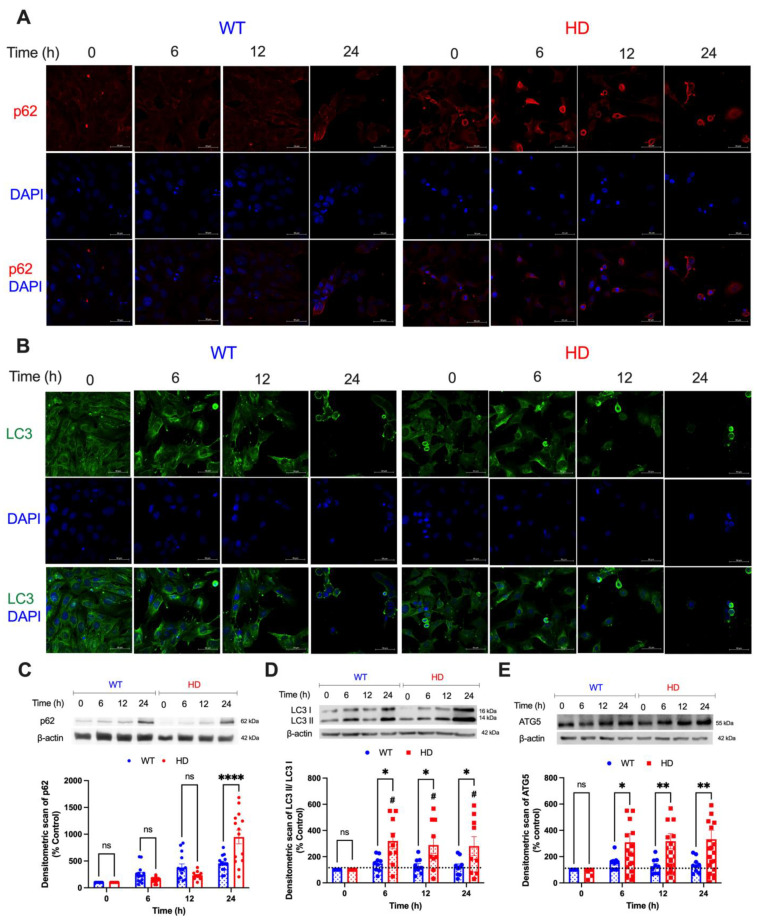
Cd upregulates autophagy-related proteins in HD striatal cells. Representative confocal images of (**A**), p62 (red), (**B**), LC3 (green), and DAPI (blue) expression in WT and HD cells upon 40 µM CdCl_2_ exposure at the indicated time points. Scale bar: 50 µm. Representative immunoblotting and quantification of (**C**) p62, (**D**) LC3-II/I, and (**E**) ATG5 in WT and HD striatal cells after 40 µM CdCl_2_ at the indicated time points. Quantitative immunoblotting data is represented as percent expression relative to the respective control within each genotype. Results are represented by the mean  ±  SEM from nine to fourteen biological replicates (N = 9–14). Sidak’s multiple comparison test compared the effect of Cd on endogenous p62, LC3, and ATG5 expression between genotypes at the indicated time points (significant differences: * *p* < 0.05, ** *p* < 0.01, and **** *p* < 0.001 for **C**–**E**). # indicates statistical significance (*p* < 0.05) compared with the respective negative controls. Images at 63× magnification.

To better understand the impact of acute Cd exposure on autophagy, we examined the protein expression of autophagic markers p62, microtubule-associated 1A/1B-light chain 3 (LC3), and autophagy-related protein 5 (ATG5) after Cd exposure at the indicated times with confocal microscopy and immunoblotting. Statistical analysis of p62 expression in both genotypes using a two-way ANOVA reveals no genotype effect (F(1, 26) = 1.809, *p* = 0.1902) and a significant time effect (F(3, 78) = 49.75, *p* < 0.0001) and exposure time (h) by genotype interaction (F(3, 78) = 15.32, *p* < 0.0001). Post hoc analysis with the Student’s *t*-test reveals a significant increase (*p* = 0.0384) in p62 expression in HD cells compared with WT only at 24 h (Figure 5C). LC3-II, a standard marker for autophagosomes, is the activated and lipidated form of LC3 and migrates faster on SDS-PAGE [61]. Cd exposure caused a significant increase in the LC3-II/I ratio in HD cells starting at baseline when compared with WT-exposed cells (Figure 5B,D). A two-way ANOVA reveals a significant effect of genotype (F(1, 16) = 6.172, *p* = 0.0244), exposure time (h) (F(3, 48) = 9.649, *p* < 0.0001), and an exposure time (h) by genotype interaction (F(3, 48) = 4.652, *p* = 0.0062). Lastly, we examined the expression of ATG5, which is important for autophagosome formation and fusion of the autophagosome with the lysosome [62]. Statistical analysis of ATG5 expression using two-way ANOVA highlights a significant effect of genotype (F(1, 16) = 9.045, *p* = 0.0083), exposure time (h) (F(3, 48) = 9.006, *p* < 0.0001), and an exposure time (h) by genotype interaction (F(3, 48) = 4.472, *p* = 0.0076), suggesting that ATG5 expression increases in proportion with the duration of Cd exposure (Figure 5E).

### 2.6. HD and Cd Cooperatively Reduce Proteasomal Activity and Augments Mitochondrial Mediated Cell Death

**Figure 6:**
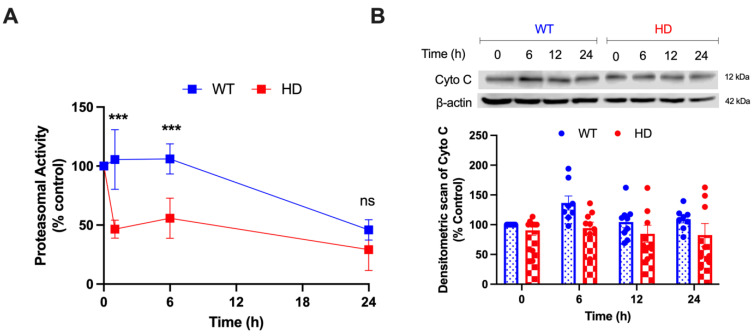
Effect of Cd exposure on UPS function and cytochrome C release. (**A**) Proteasomal activity was measured in WT and HD cells using a specific fluorogenic peptide substrate after exposure to 40 µM CdCl_2_ at the indicated time points. Proteasomal activity was normalized by the protein concentration and expressed as the percentage of vehicle-treated control cells. Results are represented by the mean  ±  SEM from eight biological replicates (N = 8). Sidak’s multiple comparison test compared the effect of Cd on proteasomal activity between genotypes at the indicated time points (significant difference: *** *p* < 0.001). (**B**) Representative Western blot of endogenous protein levels of cytochrome C (Cyto. C) after exposure to 40 µM CdCl_2_ for 0, 6, 12, and 24 h. Quantitative immunoblotting data is represented as percent expression relative to the respective control within each genotype. Results are represented by the mean  ±  SEM from four biological replicates (N = 4).

Impairment of the UPS is a common pathogenic mechanism of neurodegenerative diseases, including HD [63,64,65]. A reduction in the proteolytic activity of the UPS has been reported in the brains and other tissues of HD patients and animal models of HD [30]. To understand the synergistic effect of HD and Cd on UPS function, we assessed the enzymatic activity of the 20S/26S proteasome using the specific fluorogenic substrate Suc-LLVY-AMC in WT and HD cells. Figure 6A shows a rapid decrease in the proteasomal activity within 1 h of 40 µM Cd exposure in HD cells. A two-way ANOVA reveals a significant effect of exposure time (h) (F(2, 14) = 75.70, *p* < 0.0001), genotype (F(1, 7) = 97.12, *p* < 0.0001), and exposure time (h) by genotype interaction (F(2, 14) = 7.595, *p* = 0.0058). Less than 50% of proteasomal activity remained after the 1 h exposure (*p* < 0.0001), indicating that Cd induces proteasomal inhibition that precedes cell death. Based on the observed Cd-induced changes in mitochondrial dynamics and function in HD striatal cells (Figure 1, Figure 2, Figure 3 and Figure 4), we examined whether Cd exposure promotes the release of cytochrome C from the mitochondria to mediate intrinsic cell death in the HD cells. Figure 6B demonstrates that 40 µM Cd exposure has no significant effect on cytochrome C protein levels.

## 3. Discussion

The present study provides evidence that acute Cd exposure reduces mitochondrial potential and perturbs mitochondrial bioenergetics and function, leading to cell death in striatal HD cells. Although Cd exposure upregulated the expression of autophagic markers, it was likely insufficient to counterbalance the reduced proteasomal activity of HD. We have previously demonstrated that chronic Cd exposure potentiates reactive oxygen species (ROS) production and caspase-mediated apoptosis in an HD cell model [13]. The current study revealed that Cd triggers cytotoxicity in a time-dependent manner, with HD cells exhibiting a significant increase in cell death compared with WT (Figure 1). HD cells have been shown to have greater Cd accumulation by 48 h of exposure compared with WT [13] which might be due to the mHTT protein increasing influx of Cd [66]. We demonstrated a significant reduction in MMP in both WT and HD cells upon Cd exposure, with HD cells having ~20% greater reduction in MMP compared with WT after 24 h exposure (Figure 2A,B). This effect may be mediated by alterations in membrane phospholipid integrity [67], leading to excessive ROS generation, lower ATP production, and permeabilization of the mitochondrial membrane [68,69]. We found that Cd exposure led to a significant increase in ROS generation and activation of caspase 9 and caspase 3, leading to the initiation of the intrinsic mitochondrial-mediated cell death pathway [13]. This progressive pathogenic cascade further confirms the time-dependent effect of Cd in HD striatal cells.

mHTT impairs mitochondrial structure and function by disrupting the balance of mitochondrial dynamics [70]. mHTT directly interacts with DRP1 to enhance its activity and drive an imbalance in mitochondrial dynamics towards fission in HD models both in vivo and in vitro [71,72]. Given the overlap in cellular mechanisms between Cd exposure and mHTT-induced mitochondrial dysfunction, we hypothesized that Cd and mHTT would cooperatively accelerate alteration in overall mitochondrial bioenergetics and health in HD cells as an early mediator of neurotoxicity and cell death. In accordance with our hypothesis, Cd exposure decreased mitochondrial membrane potential in the HD cells compared with WT at 24 h (Figure 2A,B). Further, as early as 6 h Cd exposure resulted in reduced cellular ATP levels in both WT and HD cells, with an accompanying significant genotype effect observed at 24 h exposure (Figure 2C). These findings support the literature indicating that mitochondrial electron transport chain (ETC), membrane permeability, and cellular ATP production are significantly impaired by Cd exposure [73] and in ST*Hdh* cells expressing mHTT [74]. Thus, a synergistic interaction between acute Cd exposure and mHTT will substantially reduce mitochondrial bioenergetics and health to cause neurotoxicity and neurodegeneration.

mHTT and Cd independently interact with DRP1 to increase mitochondrial fission rates [70]. We observed no significant changes in DRP1 expression following Cd exposure in HD (Figure 3A,B), suggesting the combination of mHTT and Cd does not drive DRP1-dependent mitochondria fragmentation and changes in overall mitochondrial health in striatal HD cells. Although we observed a non-statistical trend of reducing DRP1 protein levels in the striatal cells at 12 h and 24 h following Cd exposure, the absence of a genotype- or time-dependent effect of Cd on DRP1 protein expression may suggest changes in overall Cd transport dynamics in the HD cells [66] that could influence DRP1 dynamics. Thus, we suspect that longer durations of Cd exposure might induce changes in DRP1 protein expression in the HD striatal cells. We observed a significant increase in FIS1 expression in wild-type cells and a non-significant trend towards a reduced expression of FIS1 in HD cells upon Cd exposure (Figure 3B,C). Cd exposure has been shown to have inconsistent effects on FIS1 expression and function in different cell types. For example, Xu et al. (2013) showed that 12 µM CdCl2 for 24 h has no effect on FIS1 protein expression in L02 liver cells [75]. Conversely, Ge et al. (2019) reported that exposure to high concentrations of CdCl_2_ (70 mg/kg) increases FIS1 mRNA expression in chicken kidney tissue [76]. It is likely that the Cd-mediated regulation of FIS1 protein expression in the striatal cells is cell type-specific and attributed to the relatively acute concentration of Cd exposure.

Mitochondrial fusion proteins play an important role in balancing mitochondrial dynamics, but there exists contradicting evidence regarding the effect of Cd exposure on pro-fusion proteins. Previous work by Ge et al. (2019) showed that exposure to higher concentrations of CdCl_2_ (70 mg/kg) caused a decrease in MFN1, MFN2, and OPA1 expression in kidney cells, while Xu et al. (2013) showed that 2 mg/kg CdCl_2_ exposure decreases MFN1, but not MFN2 or OPA1 levels in liver cells [75,76]. Due to the altered mitochondrial bioenergetics in striatal HD cells following Cd exposure (Figure 2), we hypothesized that pro-fusion proteins would experience a dramatic decrease following Cd exposure. We observed a significant decrease in MFN1 protein levels between WT and HD cells, with HD cells experiencing substantially lower levels at 6, 12, and 24 h exposure (Figure 4A,B). Additionally, we observed reduced MFN2 protein levels in HD cells than WT at 12 and 24 h Cd exposure (Figure 4A,C). These results come in agreement with Shirendeb et al.’s (2011) findings that demonstrated that expression of mHTT drives the reduction of MFN1 and MFN2 protein levels [77]. Altogether, we demonstrate that Cd, in tandem with mHTT, enhances mitochondrial fission and fragmentation by down-regulating pro-fusion proteins. 

To further understand the process of mitochondrial and protein degradation and recycling, we examined proteasomal activity and autophagic flux. Although proteasomal dysfunction in polyglutamine disorders remains not fully understood, there is sufficient evidence that mHTT sequesters components of the UPS to impair its normal function [78,79]. Recognizing the reduced proteasomal function in HD models and postmortem tissues and the negative impact of Cd-triggered oxidative damage on proteasome function [17,80,81], we hypothesized that Cd exposure and mHTT would synergistically reduce the activity and function of the UPS. Our results depict that HD cells exhibit a significantly lower activity of UPS than WT cells as early as 1 h after Cd exposure (Figure 6A). By 24 h, WT and HD cells exhibited a comparable reduction in proteasome activity, which might be due to the active inhibition of UPS caused by prolonged Cd exposure.

p62 is a key mediator of crosstalk between UPS and autophagy [82]. Given the important role autophagy plays in regulating mitochondrial homeostasis through mitophagy [83], we hypothesized that p62 would be significantly upregulated, leading to the activation of autophagy as a result of impaired mitochondrial health and UPS function. We found that Cd triggered a time-dependent increase in p62 levels in WT and HD cells, with HD cells exhibiting a significant increase at 24 h (Figure 5A–C). To further evaluate autophagy, we measured the expression of the lipidated form of LC3-II to LC3-I and found a significant increase in LC3-II/I level in HD cells over time (Figure 5A,B,D). High LC3 levels are associated with severe HD symptomatology and increased levels of downstream abnormal protein accumulation due to inefficient cargo trafficking [84]. This suggests that Cd, in tandem with HD, spurs protein misfolding and accumulation. Cd triggered a significant increase in ATG5 protein levels in HD cells as early as 6 h (Figure 5E), which is consistent with the observed increased autophagy (Figure 5A–D). Given the enhanced cell death in HD cells over time despite the activation of the autophagic pathway, it is likely that increased autophagy upon Cd exposure in striatal HD cells is insufficient to compensate for the reduced UPS function (Figure 6A), resulting in mitochondrial-mediated neurotoxicity and cell death in the striatal HD cells.

**Figure 7:**
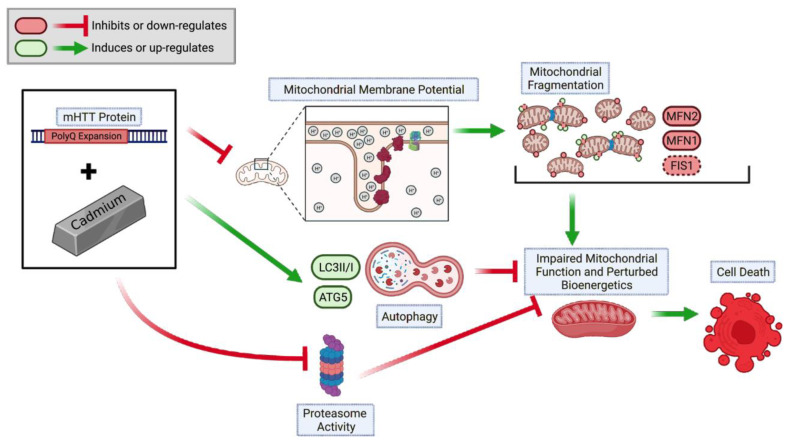
A proposed model describing the pathogenic sequence of cell death signaling events in Cd-triggered deficits in mitochondrial health and function and protein degradation pathways. HD cells at baseline exhibit reduced mitochondrial biogenesis. HD cells, in tandem with Cd exposure, exacerbate perturbations in MMP, inhibit proteasomal activity, and augment autophagic pathways. Prolonged maintenance of these pathogenic mechanisms leads to defective mitochondrial function-mediated cell death.

Cytochrome C release from the mitochondria activates the formation of the apoptosome complex by recruiting apoptotic peptidase activating factor 1 (Apaf1) and caspase-9 and further activating a caspase cascade resulting in cell death [85]. The lack of genotypic and time-dependent changes in Cd-triggered release of mitochondrial release of cytochrome C is likely due to a masking effect of cytoplasmic and mitochondrial cytochrome C levels in the striatal cells (Figure 6B). Future studies will examine cytoplasmic vs. mitochondrial cytochrome C levels in the HD striatal cells following acute exposure. We propose a sequential model of Cd-triggered neurotoxicity in HD striatal cells wherein Cd (i) selectively altered mitochondrial bioenergetics and function, decreased UPS dysfunction leading to (ii) increased ROS production, (iii) activation of apoptotic and autophagic pathways, and eventually (iv) neurotoxicity and cell death (Figure 7). This study supports and complements our previous chronic Cd and HD cytotoxicity and cell death in the ST*Hdh* neuronal model of HD [13] by elucidating the impact of acute Cd and mHTT on mitochondrial bioenergetics, dynamics, and function, as well as protein degradation pathways and cell repair pathways. Altogether, our findings will enhance the current understanding of gene–environment interactions in HD and may provide insights into practices to overcome the effects of environmental toxicants amongst at-risk populations where such chemicals and neurodegenerative diseases are prevalent.

## 4. Materials and Methods

### 4.1. Chemicals and Reagents

Cadmium (II) chloride (CdCl_2_) used in the indicated assays was purchased from Alfa Aesar (Ward Hill, MA, USA). SYTOX Green, Triton X-100, paraformaldehyde (PFA), and JC-1 probe and Hoechst dyes were purchased from Life Technologies (Grand Island, NY, USA). Hypotonic buffer (10 mM HEPES, 5 mM MgCl_2_, 10 mM KCl, 1% sucrose, and 0.1% CHAPS) components were purchased from Sigma-Aldrich (St. Louis, MO, USA). Running buffer (10X ris/Glycine/SDS Buffer), transfer buffer (10X Tris/Glycine), and nitrocellulose membrane were purchased from Bio-Rad (Hercules, CA, USA). Bovine serum albumin was purchased from ThermoFisher Scientific (Waltham, MA, USA).

### 4.2. Cell Culture

ST*Hdh*^Q7/Q7^ (WT) and ST*Hdh*^Q111/Q111^ (HD) immortalized-murine striatal-derived cells (ST*Hdh*) originally generated by Dr. Marcy MacDonald [86] were obtained from the Coriell Cell Repository (Camden, NJ, USA). Cells were cultured in Dulbecco’s modified Eagle’s medium (DMEM) (high-glucose, Sigma; D6546) supplemented with 4.5 g/L glucose and 10% fetal bovine serum (FBS) (Atlanta Biologicals; Flowery Branch, GA, USA), 2 mM GlutaMAX (Life Technologies, Inc., Carlsbad, CA, USA), 1% penicillin/streptomycin (15140-122; Life Technologies, Inc.), 0.4 mg/mL G418 sulfate (Life Technologies, Inc.), 1X nonessential amino acid solution (Life Technologies, Inc.), and 14 mM HEPES (Life Technologies, Inc.) for 16–24 h (h) prior to the assay. HD and WT cells were cultured using the above specifications and allowed to proliferate until a cell density of 85–95% was reached in a 10 cm^2^ poly-lysine-treated plate. Upon reaching 85–95% confluence, HD and WT cells were plated at 15,000 and 20,000 cells per well, respectively, in a 96-well poly-lysine-treated plate and allowed to incubate for 16–24 h before exposure to Cd. Media was replaced every three days during the incubation period. Cells were dissociated using a 0.05% trypsin/EDTA solution (Life Technologies, Inc.). Cell cultures were maintained in a humidified atmosphere of 5% CO_2_ at 33 °C, as previously described [37]. We and others have extensively used the ST*Hdh* cells as a model to study the neurotoxic mechanisms pertaining to HD [15,87,88,89,90,91]

### 4.3. Cell Death Assay

Cd-induced cytotoxicity was measured using an SYTOX Green assay, a membrane-impermeable dye that only permeates the compromised plasma membranes of dead cells and binds to nucleic acids, as described previously [92]. The fluorescence intensity is directly proportional to the number of dead cells. WT and HD striatal cells were seeded overnight and loaded with 1 µM SYTOX Green dye and allowed to incubate with 40 µM CdCl_2_ for 24 h. Cells were co-stained with Hoechst staining solution prepared by diluting a Hoechst stock solution (1:2000) in PBS, followed by 10 min incubation at room temperature, and protected from light. Changes in SYTOX Green dye and Hoechst fluorescence in the cells were visualized at 10× magnification using an EVOS microscope (Thermo Fisher Scientific, Waltham, MA, USA) and quantified at Ex/Em 549/575 and Ex/Em 350/461 nm, respectively, with a Synergy HT multimode microplate reader (BioTek Instruments, Inc., Charlotte, VT, USA).

### 4.4. Mitochondrial Membrane Potential

Mitochondrial membrane potential (MMP) was determined using a fluorescent ratiometric JC-1 dye, a lipophilic cationic probe that can selectively enter the mitochondria. Double staining of JC-1 fluorescence (red J-aggregates and green monomers) was used to assess MMP. Following seeding overnight, followed by exposure to 40 µM CdCl_2_ for the indicated times, cells were washed using 1X PBS and incubated with 5 μg/mL JC-1 for 15 min at 33 °C in the dark, then washed with 1X PBS. Cells were co-stained with Hoechst staining solution prepared by diluting a Hoechst stock solution (1:2000), as previously described. Qualitative fluorescence images of Hoechst stain (Ex/Em 350/461 nm) and red J-aggregates (Ex/Em 585/590 nm)/green monomers (Ex/Em 510/527 nm) were captured at 20× using an EVOS microscope (Thermo Fisher Scientific) and a Synergy HT Microplate.

### 4.5. ATP Assay

Cellular ATP was measured using a CellTiter-Glo. 2.0 assay (Promgea, Madison WI, USA) that provides a homogeneous method to quantify the amount of ATP present in metabolically active cells. WT and HD cells were seeded from 20,000 and 30,000 cells per well, respectively, in a solid white 96-well microplate for 16–24 h at 33 °C and 5% CO_2_. Background control wells contained medium only. Cells and controls were quadruplicated at 100 μL per well. Cells were exposed to 40 µM CdCl_2_ for 0, 6, 12, and 24 h. After Cd exposure, the microplate was equilibrated to room temperature for 30 min, and 100 μL of CellTiter-Glo 2.0 reagent was added to each well. The microplate was rocked on a plate shaker for 5 min to induce lysis and allowed to incubate at room temperature for 10 min to stabilize the luminescent signal before the Synergy HT Microplate was used to measure changes in luminescence in the cells, which is directly proportional to cellular ATP. 

### 4.6. Immunocytochemistry (ICC)

Sequential immunocytochemistry was performed to evaluate the effect of Cd exposure on protein expression and cellular localization of specific proteins. WT and HD cells were seeded at 40,000 and 50,000 cells, respectively, and incubated overnight at 33 °C in 8-well-chambered slides. Following exposure to 40 µM CdCl_2_ for the indicated time points, cells were fixed with 4% PFA for 30 min, washed with 1X PBS for 5 min three times, permeabilized with 0.01% Triton X-100 for 10 min, washed three times with 1X PBS for 5 min each, and blocked with 3% BSA for 30 min. Following blocking, cells were incubated with the first primary antibody in 3% BSA overnight at 4 °C and shaken at 2.5 rpm. Primary antibodies (ABclonal rabbit monoclonal anti-dynamin related protein (DRP1, A2586), mitochondrial fission protein 1 (FIS1, A19666), mitofusin 1 (MFN1, A9880), mitofusin 2 (MFN2, A12771), light chain 3-I/II (LC3-I/II, A5618), p62 (A19700), autophagy-related 5 (ATG5, A0203), and cytochrome C (Cyto. C, A13430)) were used at 1:100. The cells were then washed with 1X PBS for 5 min three times and incubated with Alexa Fluor 594- or 488-conjugated secondary antibodies for 1 h. For the second round of staining, cells were washed with 1X PBS for 5 min three times and re-blocked in 3% BSA for 30 min and subjected to the above staining procedure for the second round of antibodies. The cells were mounted in an antifade mounting medium (Aqua-Poly/Mount, Polysciences, Warrington, PA, USA, 18606-20) containing DAPI (VWR, IC15757410; 1:1000). Fluorescent images were acquired using a Leica DM4000B fluorescent microscope or a Zeiss LSM 880 confocal microscope at 63× magnification. Each fluorescent channel was captured in a Z-series of 6 slices to eliminate the detection of bleed-through and other artifactual fluorescence.

### 4.7. Immunoblotting

Equal numbers of WT and HD striatal cells were seeded and treated with 40 µM CdCl_2_ for the indicated times. Whole-cell lysates were prepared using RIPA buffer, 1x protease, and phosphatase inhibitor cocktails (PIA32959, Thermo Fisher Scientific) and loaded by equal protein for SDS-PAGE. The DC protein assay reagent was used to measure protein concentration in the samples. Cell lysates containing equal amounts of protein were separated on a 4–25% SDS-polyacrylamide gel. After separation, proteins were transferred to a nitrocellulose membrane, and non-specific binding sites were blocked with 3% BSA for 1 h. The membranes were incubated overnight at 4 °C with primary antibodies obtained from Abclonal and directed against DRP1 (A2586), FIS1 (A19666), MFN1 (A9880), MFN2 (A12771), LC3 (A5618), p62 (A19700), ATG5 (A0203), cytochrome C (A13430), and β-Actin (AC026) at 1:1000. Appropriate secondary antibodies from Vector Laboratories (West Grove, PA, USA) at 1:10,000 were used accordingly. Blots were visualized with Thermo Scientific Pierce Supersignal West Dura Extended Duration Chemiluminescent Substrate (Waltham, MA, USA) on the ChemiDoc Touch imager (BioRad, Hercules, CA, USA). Measurements of the integrated density of protein bands were performed using ImageStudioLite software, version 5.2.5 (LI-COR, Lincoln, NE, USA).

### 4.8. Proteasomal Activity Assay

The proteasomal peptidase assay was performed as described before [93]. After seeding WT and HD cells at 0.8 × 10^6^ and 1 × 10^6^ cells/cm^2^ per plate, respectively, overnight followed by 40 µM CdCl_2_ exposure for the indicated times, cells were harvested, washed once in 1X PBS, and lysed in hypotonic buffer (10 mM HEPES, 5 mM MgCl_2_, 10 mM KCl, 1% sucrose, and 0.1% CHAPS). Lysates were incubated with a fluorogenic substrate Suc-LLVY-AMC (75 μM) in a proteasomal assay buffer (50 mM Tris-HCl, 20 mM KCl, 5 mM MgOAc, and 10 mM dithiothreitol, pH 7.6) at 37 °C for 30 min. The cleaved fluorescent products (AMC) were examined at 380 (excitation) and 460 nm (emission) wavelengths using a fluorescence multimode plate reader (Bio-Rad). Lysate concentration in the samples was determined by the Bradford method and used to normalize proteasomal enzymatic activity. Change in proteasomal activity was expressed as fold change above baseline.

### 4.9. Statistical Analysis

All analyses were performed using Prism 9.4.1 software (GraphPad Holdings, LLC, San Diego, CA, USA) using two-way analysis of variants (ANOVA) and post hoc Dunnett’s multiple comparisons or Sidak’s multiple comparisons test unless otherwise stated. Error bars are expressed as standard errors of the mean (SEM). The significance level for all the analyses is *p* ≤ 0.05.

## Data Availability

Not applicable.
